# 5-Aminolevulinic acid promotes arachidonic acid biosynthesis in the red microalga *Porphyridium purpureum*

**DOI:** 10.1186/s13068-017-0855-4

**Published:** 2017-06-26

**Authors:** Kailin Jiao, Jingyu Chang, Xianhai Zeng, I-Son Ng, Zongyuan Xiao, Yong Sun, Xing Tang, Lu Lin

**Affiliations:** 1 College of Energy, Xiamen University, Xiamen, 361102 People’s Republic of China; 2 Xiamen Key Laboratory of High-valued Conversion Technology of Agricultural Biomass, Xiamen University, Xiamen, 361102 People’s Republic of China; 3 Department of Chemical Engineering, National Cheng Kung University, Tainan, 701 Taiwan, ROC; 4 Department of Chemical and Biochemical Engineering, College of Chemistry and Chemical Engineering, Xiamen University, Xiamen, 361005 People’s Republic of China

**Keywords:** *Porphyridium purpureum*, Fatty acid, Arachidonic acid, 5-Aminolevulinic acid, Lipidomics

## Abstract

**Background:**

The microalga *Porphyridium purpureum* within Rhodophyta abundantly produces several valuable proteins, polysaccharides, pigments and long-chain polyunsaturated fatty acid; it is especially effective in accumulating arachidonic acid (ARA). However, this high ARA yield is always achieved in conditions unfavourable for cell growth. In this study, we present a method for obtaining desirable ARA levels from *P. purpureum* while simultaneously promoting cell growth using appropriate concentrations of the growth hormone 5-Aminolevulinic acid (5-ALA).

**Results:**

Both the biomass and the ARA content of *P. purpureum* were enhanced by stimulation with 20 mg/L 5-ALA, leading to an optimal ARA yield of 170.32 mg/L—a 70.82% increase compared with control conditions. This ARA yield is the highest ever reported for microalgae. Based on variations in the fatty acid composition, total lipids, total proteins, total carbohydrates and pigment content during the cultivation period, we propose that the accumulation of ARA stimulated by 5-ALA occurs at the expense of other UFAs and total proteins, which may be related to decreased zeaxanthin. Lipidomic analysis revealed that triacylglycerols (TAGs) accounted for 47.5 ± 3.6% of all detected lipids, followed by phosphatidylglycerol (PG) and digalactosyldiacylglycerol (DGDG). As the levels of the most abundant TAGs increased under 5-ALA promotion and because 78.1 ± 3.4% (by weight) of detected TAG-branched chains contained ARA, the increase of ARA was mainly caused by TAG accumulation.

**Conclusions:**

This work demonstrated a simple and effective strategy to promote both biomass and ARA yield in *P. purpureum* by introducing a small amount of 5-ALA. These results are helpful for understanding the microalgae metabolic pathways affected by phytohormones and for guiding the development of bioproducts from microalgae.

## Background

Arachidonic acid (ARA, C20:4ω6), a long-chain polyunsaturated fatty acid (LC-PUFA), is of great nutritional importance because it is one of the major components of brain membrane phospholipids and a precursor of numerous eicosanoids. Mammals, including humans, cannot synthesize ARA directly [1]. Major ARA sources include marine fish oil, animal tissues and fungi [2]. However, shortages of these resources and the increasing interest in and demand for ARA and other LC-PUFAs have inspired the search for new sources of these PUFAs.

Although LC-PUFAs of the ω3 family, such as eicosapentaenoic acid (EPA, 20:5ω3) and docosahexaenoic acid (DHA, 22:6ω3), are abundant in several microalgae, the red unicellular rhodophyte *Porphyridium purpureum* is the only microalga reported to produce ARA in significant quantities [3, 4]. The major PUFA in this alga under favourable conditions is EPA, but under stress conditions (e.g. decreased light intensity, suboptimal temperature, suboptimal pH, increased salinity or decreased micronutrients), ARA can account for as much as 40% of total fatty acids [4–7]. In other rhodophytes, such as *Gracilaria* sp., the ARA content can reach up to 60% of the total fatty acid content [4, 8]. However, these high ARA levels usually come at the cost of reduced dry cell weight yields, which limit the ultimate yield of ARA and lower the yield of other useful components.

Phytohormones are derived from plants and affect plant growth and metabolism [9, 10]. Phytohormones, including auxin, auxinabscisic acid (ABA), cytokinin (CK), ethylene (ET), gibberellins (GAs) and 5-Aminolevulinic acid (5-ALA), have been detected in a variety of microalgal lineages. Molecular evidence from *Nannochloropsis oceanica* has demonstrated the availability of endogenous ABA and CK with physiological effects similar to those seen in higher plants [10]. However, the functional role of phytohormones in microalgae remains largely underinvestigated. Previous studies have shown that appropriate levels of phytohormones in microalgal culture could promote the production of biomass, biodiesel or selected fatty acids, whereas high concentrations can inhibit production [9]. Thus, optimizing phytohormone levels is necessary to maximize the opportunities for exploiting phytohormones for biotechnological purposes.

Recently, efforts have been made to increase the accumulation of ARA in microalgae by optimizing cultivation conditions. However, most of these studies have focused on green algae, such as *Parietochloris incise* [11] and *Myrmecia incisa* H4301 [1, 12]; few studies have focused on improving ARA synthesis from *P. purpureum* [6, 7]. In this study, we attempted to promote the lipid accumulation and ARA fraction in *P. purpureum* by optimizing cultivation conditions using phytohormones. We performed a lipidomic analysis, which has been advanced by developments in liquid chromatography mass spectrometry and chemometric methods [13], to determine the composition of lipids in *P*. *purpureum* under phytohormone stimulation.

## Methods

### Culture system

The microalga *Porphyridium purpureum* CoE1 utilized in the present study was screened and maintained by the authors’ research group. Microalga cultivation was conducted in 1000 mL flasks containing 500 mL of artificial seawater medium (ASW) with continuous illumination at a light intensity of 165 µmol/m^2^ s, a temperature of 25 °C, an initial pH of 7.6 adjusted by Tris–HCl buffer and an aeration of 1 L/min. 5-ALA was chosen as the phytohormone in the present research. In a preliminary experiment, a 5-ALA gradient of 0–20 mg/L revealed that the 10 mg/L group showed the highest biomass, whereas the 20 mg/L group showed the highest ARA content. Thus, the dosages of 5-ALA used were 0, 10, 20, 30, 40 and 50 mg/L. Each experiment was performed using an initial biomass dry weight concentration of approximately 0.4 g/L. All experiments were performed in triplicate. Cell biomass, photosynthetic pigments, fatty acid content, lipid and protein composition were tested every 2–4 days.

### Measurement of algal cell concentration

The microalgal cell dry weight (DW) per litre (g/L) was measured every 48 h according to a previously reported method (American Public Health Association, 1998). The optical density (OD_604 nm_) of the cultures was measured using a Shimadzu UV-1750 spectrophotometer. The relationship between the cell dry weight and the OD_604_ values of the algae is described by Eq. (1)

1 $${\text{DW}}\;\left( {\text{g/L}} \right) = 2.4951 \times {\text{OD}}_{604} - 0.5121\quad \left( {R^{2} = 0.997} \right)$$

where DW (g/L) is the cell dry weight concentration. This calibration curve was obtained according to a previous study [7]. The dry biomass weight was obtained by washing the cells twice with distilled water and drying them in an oven at 80 °C overnight until a constant weight was achieved.

### Analysis of lipids and fatty acids

Lipid extraction for fatty acid analysis was conducted according to Bligh and Dyer’s method [14]. Briefly, ~0.1 g of freeze-dried algae samples was added into a chloroform (2.0 mL)–methanol (1.0 mL)–water (0.8 mL) solution. Before and after an additional 2.0 mL of chloroform and 2.0 mL of water were added, the solution was intensely vibrated for 2 min. Lipids were extracted in the substratum chloroform phase after the mixture was centrifuged at 8000*g* for 10 min. The chloroform phase was then transferred into a round-bottom flask and dried under a nitrogen stream. The powdered lipids were esterified directly with 2 mL of 1 M KOH–methanol solution to obtain fatty acid methyl esters (FAMEs). Five millilitres of cyclohexane containing ~0.15 g/L C17:0 ester as internal standard was added to the solution. Subsequently, the mixture was heated at 70 °C for 40 min with a reflux condenser and then cooled and extracted with 2 mL of pure water. The upper layer was separated for GC–MS analysis.

For the FAME composition analysis, 1 μL of sample was injected into a Thermo Fisher Trace 1300-ISQLT GC–MS system equipped with an electron impact ionization detector and a TR-5MS column (30.0 m × 250 μm × 0.25 μm). The temperature of the injector and detector, the column flow rate and the split ratio were 250 °C, 1.2 mL/min and 1:50, respectively. The running temperature was set as follows: 40 °C for 1 min, heating to 230 °C at 20 °C min^−1^, held at 230 °C for 1 min, heating to 270 °C at 3 °C min^−1^ and held at 270 °C for 2 min. The quantification of FAMEs was performed by an internal standard method according to the following equation:

2 $${\text{FA}}\;{\text{content}}\;\left( {\text{mg/g}} \right) = \frac{{{\text{FA}}\;{\text{weight}}\;({\text{mg}})}}{{{\text{Alga}}\;{\text{biomass}}\;{\text{weight}}\;({\text{g}})}}$$

### FTIR analysis

Fourier transform infrared (FTIR) analysis was conducted according to a previously reported method [15]. Briefly, FTIR-attenuated total reflectance (ATR) spectra were collected on a PerkinElmer Spectrum Two instrument equipped with a diamond crystal iATR reflectance cell with a DTGS detector scanning over the wavenumber range of 4000–450 cm^−1^ at a resolution of 4 cm^−1^. Approximately 3–5 mg of finely powdered freeze-dried biomass prepared in the same manner as for the lipid and fatty acid analysis described above was applied to the surface of the crystal and then pressed onto the crystal head. Triplicates (each consisting of an average of 8 scans) for each sample were performed, and the results were averaged. Background correction scans of ambient air were made prior to each sample scan. Scans were recorded using the spectroscopic software Spectrum (version 10. PerkinElmer, Germany). Spectra were background corrected for ambient air. Ethanol was used to clean the diamond ATR between samples.

### Measurement of photosynthetic pigments

Samples (5 mL) for pigment analysis were filtered onto 25 mm diameter Whatman GF/F filters under gentle vacuum (<150 mmHg). The filters were wrapped with aluminium foil and stored in the freezer (−80 °C) until analysis. The pigment concentrations were detected by high-performance liquid chromatography (HPLC) according to the method presented by Furuya et al. [16] with some modification. The frozen filter was soaked in 1 mL N,N-dimethylformamide (DMF) in a freezer (−20 °C) for 1 h. After the extracts were centrifuged to remove cellular debris and glass fibres, the clear extract was mixed with an ion-pairing solution (1 M ammonium acetate) in a 1:1 proportion. This mixture was injected into an HPLC system with a 3.5 μm Eclipse XDB C_8_ column (100 × 4.6 mm; Agilent Technologies). The HPLC system consisted of a Shimadzu LC-20A pump with a low-pressure gradient unit FCV-20AL, an on-line degasser DGU-3A and a photodiode array UV–Vis detector SPD-M20AV with a wave-length resolution of 1.2 nm. Solvent A was 80:20 methanol:1 M ammonium acetate, and solvent B was methanol. The linear gradient was as follows (min, solvent A %, solvent B %): (0, 100, 0), (16, 45, 55), (27, 0, 100), (32, 0, 100), (40, 100, 0). The flow rate was maintained at 1 mL/min. The peaks were identified based on their retention time and absorption spectrogram compared with those of pure standards purchased from Danish Hydraulic Institute (DHI) Water and Environment, Hørsholm, Denmark.

### Lipid analysis and quantification by LC-mass/mass spectrometry

Lipids were extracted with chloroform:methanol (2:1, v/v) from ~0.1 g lyophilized algal cells. Lipidomic analysis was performed with LC-Mass/Mass Spectrometry (LC–MS/MS) using an Agilent 6490 QQQ system equipped with an Agilent 1290 Infinity LC system according to the method of Wang et al. [17]. Specifically, chloroplast membrane lipids, including PG, DGDG, monogalactosyldiacylglycerol (MGDG) and sulfoquinovosyl diacylglycerol (SQDG), were identified by precursor ion scanning for lipid ions, which yielded the diagnostic ions associated with their head groups induced by collision [18]. Phospholipids, including phosphatidylinositol (PI), phosphatidylcholine (PC) and phosphatidylethanolamine (PE), were identified according to collision-induced dissociation principles developed for these lipids [19]. The ion [C_10_H_22_NO_5_] (*m/z* 236) was used for precursor ion scanning to identify betaine lipid diacylglycerol-*O*-(N,N,N-trimethyl)-homoserine (DGTS). Sequential neutral loss scanning [20] and product ion scanning were employed to identify TAGs and fatty acyl groups, respectively. For quantitative analysis, the protonated forms ([M+H]^+^) of DGTS, PC and PE were detected by multiple reaction monitoring (MRM) in a positive ion mode, whereas the ammonium adducts ([M+NH_4_]^+^) of MGDG, DGDG and TAG were analysed by single-stage mass spectrometry (MS). The deprotonated forms of the anionic glycerolipids PG, PI and SQDG were analysed by MRM in a negative mode. The MS parameters were set as follows: nebulizing gas (nitrogen), 40 psi; dry gas (nitrogen), 4 L/min at 200 °C; spray capillary voltage, 4000 V for positive ion mode and 3500 V for negative ion mode; gas temperature, 250 °C; gas flow, 5 mL/min and sheath gas temperature, 350 °C. Prior to MS analysis, lipid extracts were separated on a ZORBAX SB C18 column (1.8 mm, i.d. 2.1 mm, length 150 mm, Agilent, USA) for the positive mode or on an Extend C18 column (1.8 mm, i.d. 2.1 mm, length 150 mm, Agilent, USA) for the negative mode. The mobile phase for the positive mode was composed of A, methanol:acetonitrile:H_2_O (19:19:2, v/v/v), and B, isopropanol; both A and B contained 10 mM ammonium acetate and 0.1% (w/v) formic acid. The following gradient was used: 0-5 min, 90% A, 10% B; 25 min, 60% A, 40% B; 45 min, 40% A, 60% B; 55 min, 20% A, 80% B; 57 min, 20% A, 80% B; 60 min, 90% A, 10% B and 70 min, 90% A, 10% B. The mobile phase for the negative mode was composed of A, methanol:acetonitrile:water (25:25:8, v/v/v) and B, isopropanol. Both A and B contained 0.025% (w/v) ammonium hydroxide. The following gradient was used: 0 min, 95% A, 5% B; 15 min, 85% A, 15% B; 25 min, 50% A, 50% B; 30 min, 37% A, 63% B; 33 min, 20% A, 80% B; 53 min, 20% A, 80% B; 53.01 min, 95% A, 5% B and 60 min, 95% A, 5% B. For each sample, the column was re-equilibrated with A for 10 min before gradient elution. The temperature of the columns was maintained at 40 °C, and the flow rate was 0.2 mL/min.

For absolute quantification, lipid extracts were mixed with internal standards (ITSDs), including TAG 17:0/17:0/17:0 (Sigma-Aldrich, USA), PE 17:0/17:0 (Avanti Polar Lipid) and PG 17:0/17:0 (Avanti Polar Lipid), PC 17:0/17:0 (Avanti Polar Lipid). Among these, PC 17:0/20:4 was used as an ITSD for both PC and DGTS quantification, and PG 17:0/17:0 was used for PG, PI and SQDG quantification. TAG 17:0/17:0/17:0 was used for MGDG and DGDG quantification. The external standards (ETSDs) for calibration included TAG 16:0/16:0/16:0 (for TAG species containing 48 carbon atoms in three acyl chains, TAG C48), TAG 16:0/18:1/16:0 (for TAG C50), TAG 18:1/16:0/18:1 (for TAG C52), TAG 18:1/18:1/18:1 (for TAG C54) (Sigma-Aldrich) and TAG 20:4/20:4/20:4 (for TAG C60) (Sigma-Aldrich). MGDG 16:3/18:3 (Matreya, USA), DGDG 18:3/18:3 (Matreya), PE 18:1/18:1 (Avanti Polar Lipid), PG 18:1/18:1 (Avanti Polar Lipid), PC 18:1/18:1 (Avanti Polar Lipid), DGTS 16:0/18:0 (Avanti Polar Lipid) and SQDG 16:0/18:3 (Avanti Polar Lipid) were used as ETSDs for the corresponding classes of membrane lipids.

## Results and discussion

### Effect of 5-ALA on cell growth

**Fig. 1:**
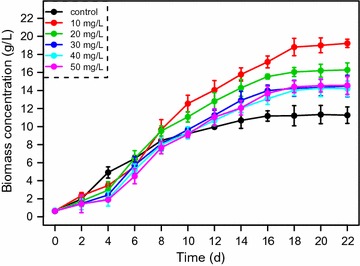
*P. purpureum* biomass concentration in the presence of different 5-ALA concentrations

The cell growth curve (Fig. 1) shows that the *P. purpureum* biomass of the control culture in the absence of 5-ALA was higher on the 4th day than the biomass of cultures including 5-ALA, but the opposite was seen beginning on the 8th day. These results suggested that *P. purpureum* benefits from 5-ALA after a short period of adaptation for the range of concentrations 10–50 mg/L and that this adaptation leads to higher final biomass yields. The highest biomass yield of 18.81 g/L was obtained with 10 mg/L 5-ALA (Table 1). This value is also the highest *P. purpureum* biomass yield ever reported. The biomass concentration was significantly enhanced with 10 and 20 mg/L 5 ALA (paired *t* test, *p* < 0.01), whereas no significant effect was found at the higher 5-ALA concentrations. Our results are consistent with those of a previous report detailing the responses of *Scenedesmus abundans* and *Chlorella ellipsoidea* to 2,4-dichlorophenoxyacetic acid (2,4-D) and/or gibberellic acid (GA3) addition [9]. The improved growth of the microalgae in the presence of phytohormones is likely due to a reduction in the level of cellular reactive oxygen species (ROS), whereas high concentrations of phytohormones may behave as herbicides [9].

**Table 1:** Biomass, pigment and fatty acid production at the 12th and 18th day

	Day 12						Day 18					
	5-ALA concentration (mg/L)						5-ALA concentration (mg/L)					
	0	10	20	30	40	50	0	10	20	30	40	50
Biomass (g/L)	9.95	14.05	12.81	11.26	10.67	11.05	11.21	18.81	16.05	14.24	14.01	14.41
Pigment concentration (g/L)												
Chl *a*	3.89	5.87	5.51	4.66	5.98	3.28	2.23	2.68	2.31	1.74	1.93	1.35
*β*-carotene	0.73	1.14	1.04	0.92	1.08	0.66	0.53	0.71	0.66	0.58	0.65	0.48
Zeaxanthin	0.99	1.14	0.78	0.82	0.94	0.89	1.17	1.06	0.69	0.80	0.62	0.60
Total	5.61	8.15	7.33	6.40	8.00	4.83	3.93	4.45	3.66	3.12	3.20	2.43
Fatty acid content (mg/g)												
ARA	5.28	1.65	4.33	4.31	3.88	2.32	8.90	7.16	10.61	8.65	5.93	5.16
EPA	5.63	1.19	3.10	3.54	3.33	1.71	7.05	3.12	4.22	3.61	2.58	2.28
TFA	30.78	16.38	26.08	25.07	26.19	23.96	51.61	33.43	46.76	40.91	34.06	30.71
UFA	18.09	8.87	14.14	17.88	14.98	12.03	33.31	20.88	27.56	24.61	18.46	17.35
UFA/TFA	58.79	54.13	54.24	71.30	57.20	50.21	64.54	62.45	58.94	60.16	54.18	56.48
AFA/TFA	17.16	10.06	16.59	17.20	14.83	9.68	17.24	21.42	22.70	21.14	17.40	16.79
ARA/EPA	0.94	1.38	1.40	1.22	1.17	1.36	1.26	2.30	2.51	2.39	2.30	2.26
Fatty acid yield (mg/L)												
TFA	306.35	230.06	333.97	282.23	279.48	264.62	578.34	628.76	750.43	582.55	477.29	442.49
ARA	52.58	23.15	55.42	48.55	41.46	25.60	99.73	134.70	170.36	123.12	83.06	74.30

### Effect of 5-ALA on fatty acids

**Fig. 2:**
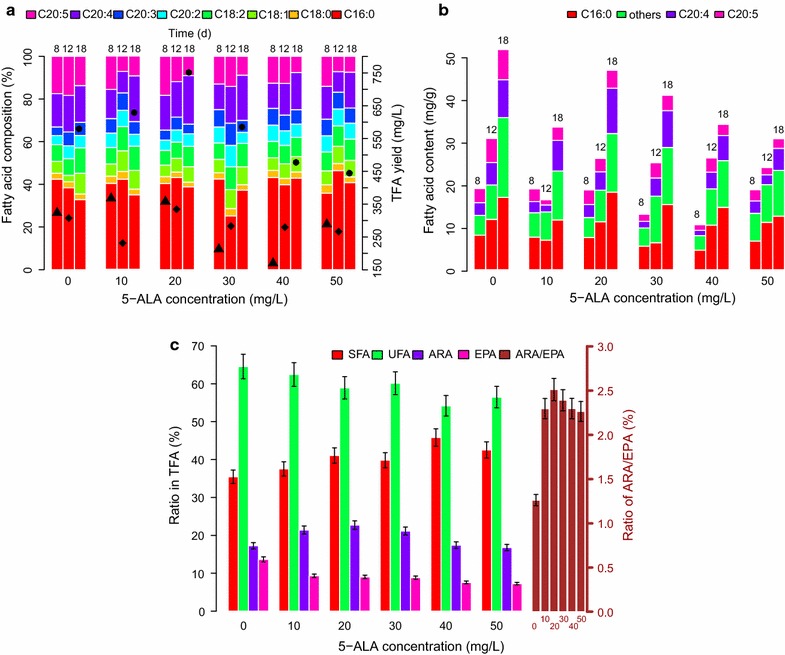
Effect of 5-ALA concentration on fatty acid content of *P. purpureum*. **a** Total fatty acid yield and fatty acid contributions. **b** Cellular fatty acid content. **c** Ratio of major fatty acid to total fatty acid

Cellular total fatty acid (TFA) content was decreased by 5-ALA addition in all cases. A maximum TFA content of 51.61 mg/g was obtained in the control group at the late exponential stage (the 18th day, Table 1). However, the negative effect of 5-ALA on the TFA in *P. purpureum* was non-linear. Among the 5-ALA addition groups, the highest TFA content (46.76 mg/g) was obtained with 20 mg/L 5-ALA (Table 1). When the concentration of 5-ALA was increased from 20 to 50 mg/L, the accumulation of TFA decreased sharply. Higher fatty acid yield occurred on the 18th day (Fig. 2a). The fatty acid yield from the groups including 5-ALA ≤20 mg/L exceeded the yield from the control (Fig. 2a; Table 1), indicating that the increase in biomass upon 5-ALA addition more than compensated for the decrease in cellular fatty acid content. The maximum fatty acid yield of 750.43 mg/L, which is 30% higher than the control (578.34 mg/L), was found with 20 mg/L 5-ALA; this yield is much higher than our previous efforts lacking phytohormones [6, 7].

During the early exponential stage, ARA content varied with no apparent regulation in any of the groups (Fig. 2b). However, ARA content increased significantly from the 12th day to the 18th day (paired *t* test, *p* = 0.03). ARA content reached its maximum of 10.61 mg/g in the 20 mg/L 5-ALA group at the 18th day and exceeded that of the control (8.90 mg/g, Fig. 2b; Table 1). A previous study reported a higher ARA content of 14.09 mg/g by optimizing the concentrations of key elements (N, P and C) in the culture medium [7]. However, with 20 mg/L 5-ALA, the cellular content multiplied by a relatively higher cell biomass yielded the highest ARA of 170.36 mg/L (Table 1), which is higher than the highest reported value of 159.74 mg/L [7]. EPA exhibited different changes compared with ARA. A significant decrease was observed in the EPA accumulation from the 8th day to the 18th day. The maximum EPA content was obtained in the control group beginning on the 12th day (Fig. 2b).

Because the greatest TFA accumulation was found on the 18th day, we further explored the fatty acid composition. The top four fatty acids were C16:0 (palmitic acid), C18:2 (linoleic acid), C20:4 (ARA) and C20:5 (EPA) (Fig. 2a). With the 5-ALA content increasing, the SFA:TFA ratio showed a slight increasing trend, whereas the ratios of UFA and EPA to TFA displayed slight decreasing trends. Both the ARA:TFA ratio and the ARA:EPA ratio exhibited maxima at 20 mg/L 5-ALA (Fig. 2c). Thus, under the influence of 5-ALA, ARA biosynthesis seems to increase at the cost of decreased levels of UFAs such as EPA. ARA and EPA are formed in ω6 and ω3 pathways from C18:2, but EPA is the final product of the biosynthesis process because the C20:4ω6 can be further desaturated to EPA by chloroplastic ∆17-desaturase [21, 22]. Thus, in the presence of 5-ALA, the newly synthesized C20:4ω6 might not be easily desaturated to C20:5ω3. Because the accumulation of ARA tends to occur in suboptimal growth conditions [5], the simultaneous increase in biomass and in the ARA/EPA ratio in the presence of 5-ALA is surprising. Thus, the mechanisms underlying these two processes may differ.

### Effect of 5-ALA on total compound content

**Fig. 3:**
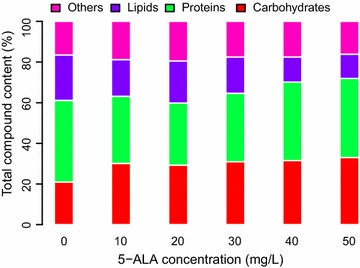
FTIR analysis of the content of total protein, carbohydrate, lipid and other species in *P. purpureum* at the 18th day

FTIR spectroscopy is a rapid and non-destructive method for the simultaneous measurement of lipid, protein and carbohydrate content in microalgal biomass [15]. To understand the variations of the *P. purpureum* bulk biochemical composition due to 5-ALA, total lipids, total proteins and total carbohydrates were analysed by FTIR, and the results are presented in Fig. 3. The changes in 5-ALA concentration affected all three biochemical compounds. Similar to the measured TFA content obtained using the traditional method, total lipids remained the highest in the control (22.40%). Total lipid accumulation was promoted in the presence of up to 20 mg/L 5-ALA; however, the values remained lower than those of the control sample. Total lipids decreased at 5-ALA concentrations greater than 20 mg/L; the highest total lipid content of 20.70% was achieved with 20 mg/L 5-ALA. The total protein content displayed a nearly inverse pattern, and the lowest protein content of 30.58% of biomass was achieved with 20 mg/L 5-ALA. Total carbohydrate content seemed to benefit from 5-ALA regardless of its concentration. The lowest carbohydrate content of 20.98% was achieved under the control conditions. These results suggested that the highest total lipid content seen with 20 mg/L 5-ALA may be assisted by the decreased biosynthesis of proteins. These proteins may include EPA-related desaturases such as ∆5-desaturase and ∆17-desaturase. However, this assumption needs further verification in future studies.

### Effect of 5-ALA on pigment

**Fig. 4:**
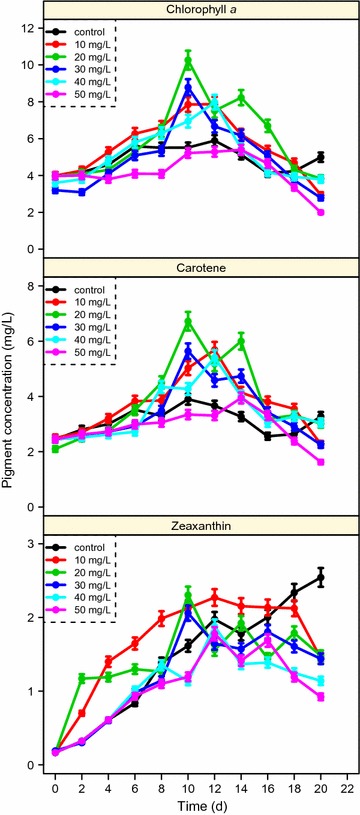
Major pigments of *P. purpureum* varied with cultivation time

Photosynthetic pigments can indicate microalgal photosynthetic efficiency. In this study, the three major pigments (excluding phycoerythrin) chlorophyll *a* (Chl *a*), zeaxanthin and *β*-carotene were detected by HPLC (Fig. 4); the levels of these three pigments were low because the most abundant pigment of *P. purpureum* is phycoerythrin [23]. No apparent differences were found among the 6 groups in terms of a changing pattern for Chl *a* and *β*-carotene, indicating no observable influence of 5-ALA on these two pigments. For zeaxanthin, there were significant differences between the experimental groups and the control group; zeaxanthin increased with time in the control group, but it decreased after the middle exponential stage in the other groups. Although the function of zeaxanthin in *P. purpureum* remains relatively contentious [24], our results indicate that the lower zeaxanthin content in the presence of 5-ALA may be related to the decreased biosynthesis of proteins and the increased accumulation of ARA.

### Quantitative analysis of glycerolipids

**Fig. 5:**
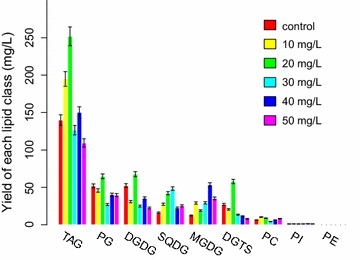
Yields of different glycerolipid classes in *P. purpureum* in the presence of different 5-ALA concentrations at the 18th day

**Fig. 6:**
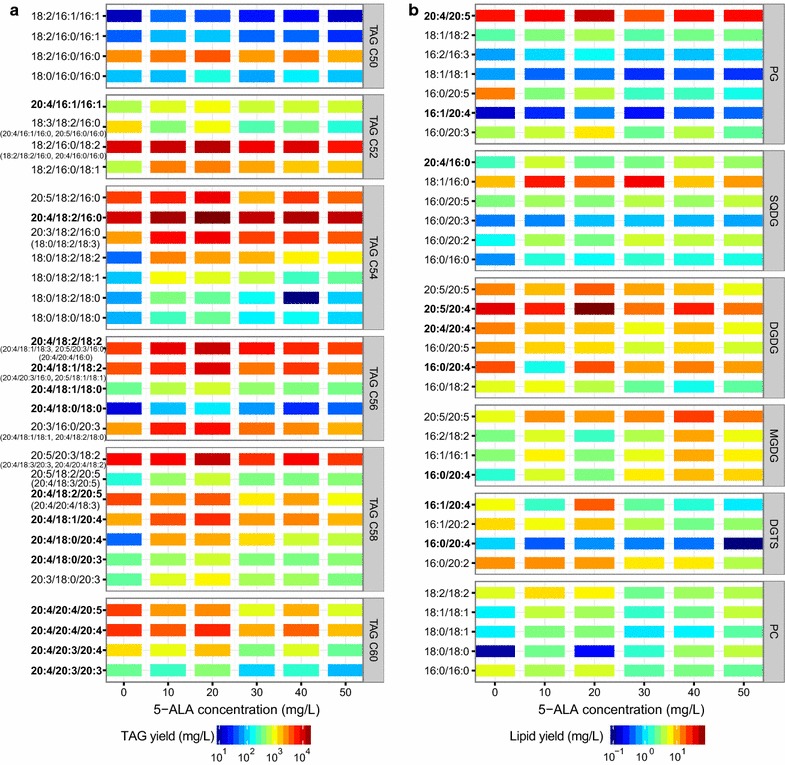
Yields of glycerolipids in *P. purpureum* with different 5-ALA concentrations at the 18th day. **a** TAG yields, **b** other major polar lipids

Lipidomics, first defined in 2003, is a branch of metabolomics used to identify all lipids and the molecules with which they interact in biological systems [25, 26]. However, no previous studies have elucidated the lipidomic changes in *P. purpureum*. Based on the LC–MS/MS analysis, TAG was the most dominant lipid in *P. purpureum* at the 18th day, accounting for 47.5 ± 3.6% of all the detected lipids (Fig. 5). We identified 47 types of TAGs, in formations of C50, C52, C54, C56, C58 and C60 (Fig. 6a). Although TAG synthesis is considered a protective strategy by which microalgae cope with environmental stress (e.g. nutrient deprivation, high light) [27, 28], our results revealed that *P. purpureum* can accumulate TAGs under favourable growing conditions (Fig. 5). In the group with 20 mg/L 5-ALA, TAGs reached their maxima for most of the dominant forms (Fig. 6a). Similarly, the accumulation of TAGs under favourable culture conditions has recently been reported in *Haematococcus pluvialis*, *Chlamydomonas* and *Nannochloropsis* [17, 29].

Additionally, we identified 8 PG, 6 DGDG, 6 SQDG, 4 MGDG, 4 DGTS, 5 PC, 4 PE and 1 PI in *P. purpureum* (Fig. 6b). Most of these lipids increased upon the addition of 5-ALA at concentrations less than 40 mg/L (Fig. 6b).

TAGs are reportedly mostly composed of saturated and monounsaturated fatty acids even in algae that are rich in polyunsaturated fatty acids (PUFAs) [30]. In this study, we found that TAGs were the most abundant lipids containing ARA and EPA, followed by PG and DGDG (Table 2). TAGs were especially rich in ARA (78.1 ± 3.4% of detected TAG-branched chains by weight), whereas PG contained more EPA than ARA (Fig. 6; Table 2). ARA reached its maximum in TAGs and DGDG upon the addition of 20 mg/L 5-ALA (Fig. 6; Table 2), which contributed the majority of ARA accumulation.

**Table 2:** ARA and EPA yields contained in TAG, PG and DGDG

5-ALA (mg/L)	TAG-all (mg/L)	20:4-TAG (%)	20:5-TAG (%)	PG-all (mg/L)	20:4-PG (%)	20:5-PG (%)	DGDG-all (mg/L)	20:4-DGDG (%)	20:5-DGDG (%)
0	140.06	84.67	45.12	51.90	58.47	86.29	52.37	70.32	62.36
10	194.68	74.93	34.97	45.54	75.68	81.04	30.99	61.99	72.68
20	251.76	77.54	38.62	64.38	74.55	79.92	67.66	77.86	75.62
30	126.15	77.35	31.28	26.72	72.83	78.31	25.12	64.10	62.14
40	149.69	78.02	37.43	39.99	76.55	79.94	35.58	72.91	63.81
50	108.79	76.02	33.22	39.38	81.51	83.76	22.55	72.73	56.74

## Conclusion

This study explored the potential effects of 5-ALA on the growth of *P. purpureum* and its biochemical compounds and was the first to analyse the lipidomics in *P. purpureum* by LC–MS/MS. The results demonstrated that with 20 mg/L 5-ALA, *P. purpureum* grew significantly better than under control conditions, leading to a biomass content enhancement of up to 43.18%. Under these conditions, the maximum fatty acids yield (750.29 mg/L), ARA yield (170.32 mg/L), cellular ARA content (10.61 mg/g) and ARA:TFA ratio (2.51) were obtained, which correspond to 29.76, 70.82, 19.21 and 99.21% increases, respectively, compared with the control. This higher ARA accumulation was associated with enhanced TAG accumulation and decreased levels of other UFAs, total protein and zeaxanthin. Zeaxanthin may play an important role in regulating the biosynthesis of metabolic products in *P. purpureum*; these pathways are distinct in stress and non-stress conditions. Further investigations should focus on the underlying mechanisms to further enhance the yields of valuable PUFAs by *P. purpureum*.

## Abbreviations

ARA: arachidonic acid
5-ALA: 5-Aminolevulinic acid
TAG: triacylglycerol
LC-PUFA: long-chain polyunsaturated fatty acid
EPA: eicosapentaenoic acid
DHA: docosahexaenoic acid
ABA: auxinabscisic acid
CK: cytokinin
ET: ethylene
GAs: gibberellins
ASW: artificial seawater medium
DW: dry weight
OD: optical density
FAMEs: fatty acid methyl esters
FTIR: Fourier Transform Infrared
ATR: attenuated total reflectance
HPLC: high-performance liquid chromatography
DMF: N,N-dimethylformamide
DHI: Danish Hydraulic Institute
LC–MS/MS: LC-mass/mass spectrometry
MGDG: monogalactosyldiacylglycerol
DGDG: digalactosyldiacylglycerol
SQDG: sulfoquinovosyl diacylglycerol
PG: phosphatidylglycerol
PI: phosphatidylinositol
PC: phosphatidylcholine
PE: phosphatidylethanolamine
DGTS: diacylglycerol-*O*-(N,N,N-trimethyl)-homoserine
MRM: multiple reaction monitoring
ITSD: internal standard
ETSD: external standard
ROS: reactive oxygen species
TFA: total fatty acid
Chl *a*: chlorophyll *a*

## References

[1] Ouyang LL, Li H, Liu F, Tong M, Yu SY, Zhou ZG, Li H, Liu F, Tong M, Dumancas GG, Murdianti BS, Lucas EA (2013). Accumulation of arachidonic acid in a green microalga, *Myrmecia Incisa* H4301, enhanced by nitrogen starvation and its molecular regulation mechanisms. Arachidonic acid: dietary sources and general functions.

[2] Gill I, Valivety R (1997). Polyunsaturated fatty acids, part 1: occurrence, biological activities and applications. Trends Biotechnol.

[3] Cohen Z (1990). The production potential of eicosapentaenoic and arachidonic acids by the red alga *Porphyridium cruentum*. J Am Oil Chem Soc.

[4] Bigogno C, Khozin-Goldberg I, Boussiba S, Vonshak A, Cohen Z (2002). Lipid and fatty acid composition of the green oleaginous alga *Parietochloris incisa*, the richest plant source of arachidonic acid. Phytochemistry.

[5] Cohen Z, Vonshak A, Richmond A (1988). Effect of environmental conditions on fatty acid composition of the red alga *Porphyridium cruentum*: correlation to growth rate. J Phycol.

[6] Su G, Jiao K, Chang J, Li Z, Guo X, Sun Y, Zeng X, Lu Y, Lin L (2016). Enhancing total fatty acids and arachidonic acid production by the red microalgae *Porphyridium purpureum*. Bioresources and Bioprocessing..

[7] Su G, Jiao K, Zheng L, Guo X, Chang J, Ndikubwimana T, Yong S, Zeng X, Lu Y, Lu L (2016). Phosphate limitation promotes unsaturated fatty acids and arachidonic acid biosynthesis by microalgae *Porphyridium purpureum*. Bioprocess Biosyst Eng.

[8] Araki S, Sakurai T, Oohusa T, Kayama M, Nisizawa K (1990). Content of arachidonic and eicosapentaenoic acids in polar lipids from *Gracilaria* (Gracilariales, Rhodophyta). Hydrobiologia.

[9] González-Garcinuño Á, Martin DV, Eva M, Galán MA (2016). Understanding and optimizing the addition of phytohormones in the culture of microalgae for lipid production. Biotechnol Prog..

[10] Lu YD, Jian X (2015). Phytohormones in microalgae: a new opportunity for microalgal biotechnology?. Trends Plant Sci.

[11] Solovchenko AE, Khozin-Goldberg I, Didi-Cohen S, Cohen Z, Merzlyak MN (2008). Effects of light intensity and nitrogen starvation on growth, total fatty acids and arachidonic acid in the green microalga *Parietochloris incisa*. J Appl Phycol.

[12] Ouyang LL, Chen SH, Li Y, Zhou ZG (2013). Transcriptome analysis reveals unique C4-like photosynthesis and oil body formation in an arachidonic acid-rich microalga *Myrmecia incisa Reisigl* H4301. BMC Genomics..

[13] Roberts LD, Mccombie G, Titman CM, Griffin JL (2008). A matter of fat: an introduction to lipidomic profiling methods. J Chromatogr, B: Anal Technol Biomed Life Sci.

[14] Bligh EG, Dyer WJ (1959). A rapid method of total lipid extraction and purification. Can J Biochem Physiol.

[15] Mayers JJ, Flynn KJ, Shields RJ (2013). Rapid determination of bulk microalgal biochemical composition by Fourier-transform infrared spectroscopy. Bioresour Technol.

[16] Furuya K, Hayashi M, Yabushita Y (1998). HPLC determination of phytoplankton pigments using N,N-dimethylformamide. J Oceanogr.

[17] Wang B, Zhang Z, Hu Q, Sommerfeld M, Lu Y, Han D (2014). Cellular capacities for high-light acclimation and changing lipid profiles across life cycle stages of the green alga *Haematococcus pluvialis*. PLoS ONE.

[18] Welti R, Wang X, Williams TD (2003). Electrospray ionization tandem mass spectrometry scan modes for plant chloroplast lipids. Anal Biochem.

[19] Hsu FF, Turk J (2009). Electrospray ionization with low-energy collisionally activated dissociation tandem mass spectrometry of glycerophospholipids: mechanisms of fragmentation and structural characterization. J Chromatogr B Anal Technol Biomed Life Sci.

[20] Han X, Gross RW (2001). Quantitative analysis and molecular species fingerprinting of triacylglyceride molecular species directly from lipid extracts of biological samples by electrospray ionization tandem mass spectrometry. Anal Biochem.

[21] Cohen Z, Shiran D, Khozin I, Heimer YM (1997). Fatty acid unsaturation in the red alga *Porphyridium cruentum*. Is the methylene interrupted nature of polyunsaturated fatty acids an intrinsic property of the desaturases?. Biochem Biophys Acta.

[22] Khozin I, Adlerstein D, Bigongo C, Heimer YM, Cohen Z (1997). Elucidation of the biosynthesis of eicosapentaenoic acid in the microalga *Porphyridium cruentum* (II. Studies with radiolabeled precursors). Plant Physiol.

[23] Safi C, Charton M, Pignolet O, Pontalier PY, Vaca-Garcia C (2013). Evaluation of the protein quality of *Porphyridium cruentum*. J Appl Phycol.

[24] Kopecký J, Lukavská A, Verboviková E, Pfündel E (2004). Changes in the photosynthetic pigment patterns during the synchronous life cycle of *Porphyridium purpureum*. Algol Stud.

[25] Han X, Gross RW (2005). Global analyses of cellular lipidomes directly from crude extracts of biological samples by ESI mass spectrometry: a bridge to lipidomics. Mass Spectrom Rev.

[26] Zehethofer N, Pinto DM (2008). Recent developments in tandem mass spectrometry for lipidomic analysis. Anal Chim Acta.

[27] Markn M, Olgab C, Olgaa G, Irinav R, Alexeie S, Inna KG, Zvi C (2007). Effect of nitrogen starvation on optical properties, pigments, and arachidonic acid content of the unicellular green alga *Parietochloris incisa* (Trebouxiophyceae, Chlorophyta). J Phycol.

[28] Solovchenko AE (2012). Physiological role of neutral lipid accumulation in eukaryotic microalgae under stresses. Russ J Plant Physiol.

[29] Liu B, Vieler A, Li C, Jones AD, Benning C (2013). Triacylglycerol profiling of microalgae *Chlamydomonas reinhardtii* and *Nannochloropsis oceanica*. Bioresour Technol.

[30] Cohen Z, Khozingoldberg I, Adlerstein D, Bigogno C (2000). The role of triacylglycerol as a reservoir of polyunsaturated fatty acids for the rapid production of chloroplastic lipids in certain microalgae. Biochem Soc Trans.

